# Determination of immune factor levels in serum and local hematoma samples of osteoporotic fracture patients and clinical study of the effect of active vitamin D3 treatment on immune factor levels

**DOI:** 10.1186/s13018-023-03777-7

**Published:** 2023-04-10

**Authors:** Sijia Liu, Jianjun Li, Mingwei Zhang

**Affiliations:** Shengjing Hospital of China Medical University, Shengjing, China

**Keywords:** Osteoporotic fractures, Immune factors, Active vitamin D3

## Abstract

**Objective:**

The aim of this study was to investigate changes in systemic and local immune factors, namely, interleukin (IL)-1β, IL-6 and tumor necrosis factor (TNF)-α, in patients with and without osteoporotic fractures and to explore the effects of active vitamin D3 treatment on immune function and fracture prognosis in patients with osteoporotic fractures.

**Method:**

The mRNA expression levels of IL-1β, IL-6 and TNF-α were measured before the operation. After the operation, the patients in the control group were treated with conventional fracture treatment and calcium supplementation, and the patients in the treatment group were treated with calcium plus active vitamin D3 in addition to conventional fracture treatment. The serum of each patient was collected on the seventh day after the operation.

**Results:**

The expression levels of the three immune factors (IL-1β, IL-6 and TNF-α) in the fracture end hematoma samples were significantly positively correlated with those in the serum samples (*P* < 0.05). The mean values of the serums of IL-1β, IL-6 and TNF-α in the osteoporosis group were significantly higher than those in the non-osteoporosis group (*P* < 0.05). The average number of hematomas in the osteoporosis group was significantly higher than that in the non-osteoporosis group (*P* < 0.05). The results for the active vitamin D3 treatment group were significantly lower than those for the control group (*P* < 0.05). The mean wrist function score of the active vitamin D3 treatment group was significantly better than that of the control group (*P* < 0.05). The average fracture healing time of the treatment group was significantly shorter than that of the control group (*P* < 0.05).

**Conclusion:**

The relative expression of IL-1β, IL-6, and TNF-α in the fracture end hematoma samples was positively correlated with the corresponding levels of these immune factors in the serum samples. The levels of IL-1β, IL-6 and TNF-α in the serum and fracture end hematoma samples of the osteoporotic fracture patients were higher than those of the non-osteoporotic fracture patients. Active vitamin D3 treatment promoted fracture healing by affecting the levels of these immune factors.

## Introduction

With the aging of the population, osteoporosis has gradually become a prominent public health problem. According to statistics, osteoporosis patients in the world have reached about 200 million people. According to the “White Paper on Osteoporosis in China” released in June 2009, the prevalence of osteoporosis in Chinese people is 9.9% and 11.1% based on the BMD measured at the vertebral body and femoral neck, respectively (17.0% and 5.8% in men and 12.2% and 15.5% in women, respectively) [1]. Increased bone fragility and decreased bone mass are the main characteristics of osteoporosis, leading to an increased risk of fracture is the main consequence of osteoporosis, and the most serious consequence of osteoporosis is osteoporotic fracture [2]. The most common sites of osteoporotic fractures are vertebral body, hip and wrist. It has been reported in Shanghai, China, that about 20% of patients with osteoporotic fracture die within one year. The causes include respiratory, cardiovascular and cerebrovascular diseases after fracture, which may be caused by long-term bedridden. In addition, more than one third of patients with hip fracture lose the ability to live independently. Therefore, osteoporotic fractures seriously threaten the life and quality of life of the elderly in the current situation of population aging. Studies have shown changes in the serum levels of immune factors, including interleukins (ILs) and tumor necrosis factor (TNF), in osteoporosis patients. Fracture is a serious trauma to the body that often occurs after a certain violent injury. In this process, the body undergoes a serious immune response, and a variety of immune factors and immune cells are involved and change to different degrees, but the mechanism of the above changes is not clear at present.

Interleukins are composed of many components, such as IL-1, which is a pro-inflammatory cytokine that increases osteoclast activity. IL-1 is synthesized by macrophages and includes two isoforms, IL-1α and Il-1β, which have been confirmed in experiments in different environments to enhance osteoclast activity [3]. Other interleukins (such as IL-4, IL-10, IL-13 and IL-18) can inhibit OC production [4]. In addition to the interleukin family, other inflammatory cytokines also have an effect on bone metabolism. Tumor necrosis factor (TNF) includes two isoforms, α and β. TNF-α can promote the activity of osteoclast (OC) and inhibit bone formation in arthritis and the elderly [5]. In addition, TNF can stimulate the p38 MAPK signaling pathway and increase the synthesis of RANKL in bone marrow stromal cells. The activity of OC was further enhanced [6]. Experiments in diabetic rats have shown that TNF-α and RANKL have relatively little effect on OC but can inhibit the occurrence of osteoblast (OB). Meanwhile, TNF-α promoted the expression of glycogen synthase kinase-3β (GSK-3β) gene. There is a balance of bone metabolism between OB and OC, and there is an interaction between TNF-α, RANKL and GSK-3β, which is likely to break this balance and lead to osteoporosis [7]. IL-6, another pro-inflammatory cytokine, has been shown to have dual effects on bone metabolism. The changes of this dual effect depend on the different pathological or physiological states of the bone in vivo, and on the differentiation process of OC in vitro. IL-6 can promote the occurrence of OC when OB coexists. When only acting on highly purified OC (i.e., in the absence of OB), IL-6 could not promote the occurrence of OC [8]. Some studies also suggest that IL-6 can directly reduce the occurrence of OC, which may be the result of the inhibition of the receptor of NK-κB signaling pathway [9].

In addition, active vitamin D3 can regulate human calcium and phosphorus metabolism, enhance immune function, and play a huge role in the treatment of osteoporosis. However, existing studies often start with the effect of vitamin D3 on calcium absorption. Whether vitamin D3 can affect bone metabolism by regulating immune function is still unclear. Studies on the effects of immune factors on bone metabolism also mostly directly use the immune factors extracted from serum for quantitative analysis, which also brings the following questions: Can serum immunological markers be used to define the local immune metabolism at the broken end of osteoporotic fracture? However, there is no clear experiment to verify whether the expression of serum immune factors is consistent with that of local hematoma at the broken end of osteoporotic fracture. The main objective of this study was to investigate the correlation between the levels of the common immune factors IL-1β, IL-6, and TNF-α in serum samples and the expression of these factors in fracture end hematoma samples and to evaluate whether active vitamin D3 can affect the prognosis of fracture patients through immune regulation of bone metabolism.

## Materials and methods

### Main materials and instruments

#### Main reagent materials

The main materials used were as follows: PCR and reverse transcription kits and TRIzol reagent (TaKaRa Company).

#### Main experimental instruments

The instruments and equipment used were as follows: a real-time quantitative PCR instrument (ABI Company), EP tubes (Nest Company), a − 80 °C deep-freeze refrigerator (Sanyo Company), a table-top and high-speed refrigerated centrifuge (Thermo Company), an ultramicro-spectrophotometer (Thermo Company), Eppendorf tubes (Gilson Company), and a clean bench (Haier Company).

### Selection of experimental data

#### Inclusion criteria for trial data

The inclusion criteria were as follows: (1) patients diagnosed with distal radius fractures treated at Shengjing Hospital of China Medical University; (2) patients treated with open reduction and internal fixation; and (3) patients over 18 years old. The Ethics Committee of Shengjing Hospital of China Medical University approved the conduct of this study (Ethics number: 2021PS041K).

#### Exclusion criteria for test data

The exclusion criteria were as follows: (1) patients with diabetes, rheumatic diseases, recent glucocorticoid therapy, pathological fracture, and other underlying diseases affecting bone metabolism; (2) patients that had been merged into composite trauma or combined with other autoimmune disease; (3) fracture patients undergoing closed reduction and patients with an onset time > 1 week during surgical treatment; and (4) patients with recent treatment with vitamin D or its analogs.

#### Collection of test data

The clinical data of patients with distal radius fractures were collected during ordinary clinical work. For patients who choose to undergo open reduction and internal fixation surgery. Bone mineral density (BMD) was measured by dual energy X-ray absorptiometry before the operation. According to the BMD T value, the patients were divided into an osteoporosis group (T ≤ − 2.5) and a non-osteoporosis group (T > − 2.5). The osteoporosis group was further divided into two subgroups (active vitamin D3 treatment group and control treatment group) by simple randomization. The serum levels of IL-1β, IL-6 and TNF-α in each patient were detected before the operation and on the seventh day after the operation. All operations were performed by the same treatment team. Fracture end hematoma tissues were collected during the operation and then sent to Benxi Experimental Base of Shengjing Hospital of China Medical University for RNA purification, reverse transcription and cryopreservation to prepare for the subsequent determination of the relative expression levels of IL-1β, IL-6, and TNF-α by real-time fluorescence quantitative PCR (qPCR). From January 2019 to November 2020, a total of 48 patients who met the inclusion and exclusion criteria, namely, 29 patients in the osteoporosis group and 19 patients in the non-osteoporosis group, were enrolled at the Department of Orthopedic Trauma, Shengjing Hospital of China Medical University. Their clinical data and hematoma specimens were collected. There were 15 patients in the active vitamin D3 treatment subgroup and 14 patients in the control treatment subgroup in the osteoporosis group.

### Treatment methods

All patients who met the inclusion and exclusion criteria were prepared according to the treatment protocol for open reduction and plate internal fixation of distal radius fractures. Preoperative cardiopulmonary function tests were completed, informed consent was signed, and BMD was measured by dual energy X-ray absorptiometry. After routine preoperative preparation, the serum levels of IL-1β, IL-6 and TNF-α were detected in the morning of the operation day. All operations were performed by the same treatment team. The fracture end hematoma tissues were collected during the operation, frozen quickly, and sent to Benxi Experimental Base in batches for further treatment. The control group was treated with conventional fracture treatment and calcium carbonate D3 tablets supplementation, and the treatment group was additionally treated with oral active vitamin D3 (Rogaquan, Switzerland Roche LTD; 0.25 μg/time, two times a day). Postoperative medication and functional rehabilitation guidance were carried out according to the unified standard. The serum levels of IL-1β, IL-6 and TNF-α were detected on the seventh day after the operation. In addition, the patients were instructed to carry out muscle and joint functional exercise immediately after the operation, and the fracture healing status was regularly reviewed by X-ray film. The patients were followed up at 6 months after the operation, and the wrist function of the patients was evaluated according to the Gartland and Werley (GW) function scoring standard.

### Specimen processing methods

1. Approximately 100 mg of tissue was removed, cut into pieces, added to 75% ethanol solution in sterile enzyme-free water, dissolved at room temperature, ground thoroughly until no large tissue could be seen, added to 1 ml of TRIzol, and allowed to stand.
2. After centrifugation at 1200 rpm for 15 min at 4 °C, the supernatant was retained, 200 µl of chloroform was added, and the mixture was thoroughly mixed.
3. The samples were centrifuged again at 1200 rpm for 15 min at 4 °C, the supernatant was transferred to a new tube, isopropanol was added to approximately four-fifths of the volume of the supernatant, and the samples were mixed and incubated at -20 °C.
4. The next day, the samples were centrifuged at 4 °C and 8000 rpm for 5 min and then washed twice. The supernatant was discarded, and the samples were blown dry for 15 min.
5. One milliliter of pure 75% ethanol was added, and after mixing, the plume suspension, that is, the extracted RNA, was collected.
6. The excess ethanol was discarded, an appropriate amount of enzyme-free water was added, and the RNA concentration and purity were determined by using an ultramicro-spectrophotometer.
7. After configuration of the reverse transcription reaction system, the reaction was performed at 37℃ for 15 min and 85℃ for 5 s, and then the reaction was allowed to stand at 4℃. At this time, the RNA reverse-transcribed cDNA was obtained and cryopreserved.
8. An ABI 7500 Fast RT PCR system was used to detect the expression of related genes. The reaction conditions were as follows: ① denaturation—the DNA was heated to 95℃ for predenaturation for 5 min and then was denatured at 95℃ for 30 s; ② DNA annealing—the temperature was lowered to 60 °C for approximately 30 s to allow base pairing of the complementary DNA template strands and primers; ③ primer extension—the target sequence and dNTPs were used as the template and raw material, respectively, and the DNA template primer became a replication strand under the action of polymerase, which is complementary to the template DNA strand; and ④ the three-step reaction was repeated, and 40 cycles of amplification were performed after 1 min of extension at 72 ℃, with the last extension at 72 ℃ for 5 min. The primer sequences were as follows:

**Table:** 

Item	Primer Sequence
IL-1β	Forward: 5′-CCAGGGACAGGATATGGAGCA-3′
	Inverse: 5′-TTCAACACGCAGGACAGGTACAG-3′
IL-6	Forward: 5′-AAGCCAGAGCTGTGCAGATGAGTA-3′
	Inverse: 5′-TGTCCTGCAGCCACTGGTTC-3′
TNF-α	Forward: 5′-CTGCCTGCTGCACTTTGGAG-3′
	Inverse: 5′-ACATGGGCTACAGGCTTGTCACT-v
β actin	Forward: 5′-CCTAAGGCCAACCGTGAAAA-3′
	Inverse: 5′-CAGAGGCATACAGGGACAACAC-3′

### Statistical methods

SPSS (Statistical Product Service Solutions) 25.0 software was used to analyze the data. The expression of immune factors was expressed as the mean ± standard deviation (|x|± sd), using monadic linear regression analysis and correlation, respectively, using the chi-square test and independent sample t test analysis of the sample mean. For all statistical methods, differences were considered statistically significant when *P* < 0.05. GraphPad Prism 9 software was used to draw statistical maps based on the statistical results. ΔCT = CT(target gene)/CT(reference gene) was used to record the relative expression of immune factors.

## Results

### General data of the included patients

Forty-eight patients, namely, 22 males and 26 females, with an average age of 59.5 ± 4.4 years, met the inclusion and exclusion criteria and were selected for this study. The osteoporosis group comprised 29 patients, namely, 12 males and 17 females, with an average age of 60.0 ± 4.2 years, an average height of 1.68 ± 0.05 m, and an average weight of 74.8 ± 7.3 kg. There were 19 patients in the non-osteoporosis group: 10 males and 9 females, with an average age of 58.7 ± 4.7 years, an average height of 1.69 ± 0.05 m, and an average weight of 76.3 ± 8.6 kg. There was no significant difference in the general data between the two groups (*P* > 0.05). In the osteoporosis group, there were 15 patients in the active vitamin D3 treatment group, namely, 6 males and 9 females, with an average age of 58.6 ± 2.8 years, an average height of 1.67 ± 0.05 m, and an average weight of 72.8 ± 8.2 kg. There were 14 patients in the control group, namely, 6 males and 8 females, with an average age of 61.4 ± 4.7 years, an average height of 1.70 ± 0.06 m, and an average weight of 76.9 ± 5.7 kg. There was no significant difference in the general data between the two groups (*P* > 0.05). See Table 1 and Table 2 for details.

**Table 1:** Comparison of the general data of the patients in the osteoporosis and non-osteoporosis groups

Group	n	Age	Sex (male/female)	Height (meter)	KG
Osteoporosis group	29	60.0 ± 4.2	12/17	1.68 ± 0.05	74.8 ± 7.3
Non-osteoporosis group	19	58.7 ± 4.7	10/9	1.69 ± 0.05	76.3 ± 8.6
Statistic		t = -0.913	X^2^=0.557	t = 0.679	t = 0.647
P		> 0.05	> 0.05	> 0.05	> 0.05

**Table 2:** The general data of the patients in the active vitamin D3 treatment and control groups

Group	n	Age	Sex (male/female)	Height (meter)	KG
Treatment group	15	58.6 ± 2.8	6/9	1.67 ± 0.05	72.8 ± 8.2
Control group	14	61.4 ± 4.7	6/8	1.70 ± 0.06	76.9 ± 5.7
Statistic		t = 0.064	X^2^ = 0.876	t = 0.231	t = 0.135
P		> 0.05	> 0.05	> 0.05	> 0.05

### Comparison of the expression levels of IL-1β, IL-6 and TNF-α in serum and fracture end hematoma samples

The expression levels of IL-1β, IL-6 and TNF-α in the serum and fracture end hematoma samples of the 48 patients were statistically analyzed, and univariate linear regression analysis was performed. The relative expression levels of each gene in the fracture end hematoma samples were used as independent variables, and serum immunological indicators were used as dependent variables to describe their correlation. The statistical results are as follows (Table 3):

**Table 3:** Serum immune factor levels and relative expression of immune factors in fracture end hematoma samples

	IL-1β		IL-6		TNF-α	
	Blood serum(ng/ml)	Hematoma	Blood serum (µg/ml)	Hematoma	Blood serum (ng/ml)	Hematoma
Average	14.68 ± 3.71	1.0054 ± 0.0480	64.16 ± 10.12	1.0045 ± 0.0652	711.38 ± 139.93	0.9993 ± 0.0538
One yuan Linear Return to the equation	y = -56.848 + 71.138x		y = -79.4121 + 142.928x		y = -1740.626 + 2453.61x	
F	289.3		254.7		372.452	
P	< 0.05		< 0.05		< 0.05	

The above data analysis results indicate that the serum level of IL-1β in patients with distal radius fracture was positively correlated with the relative expression of IL-1β in the fracture end hematoma samples; the correlation coefficient was 71.138, and the regression was statistically significant (*P* < 0.05). The serum IL-6 level was positively correlated with the relative expression of IL-6 in the fracture end hematoma samples; the correlation coefficient was 142.928, and the regression was statistically significant (*P* < 0.05). The serum TNF-α level was positively correlated with the relative expression of TNF-α in the fracture end hematoma samples; the correlation coefficient was 2453.61, and the regression was statistically significant (*P* < 0.05).

### Changes in immunological indexes in osteoporotic fracture patients compared with non-osteoporotic fracture patients

According to the BMD T value measured by dual energy X-ray absorptiometry before the operation, 48 patients were divided into an osteoporosis group (T < -2.5, 29 cases) and a non-osteoporosis group (T > -2.5, 19 cases). The levels of IL-1β, IL-6 and TNF-α in the serum and fracture end hematoma samples of the two groups were recorded, and their means were compared by independent sample t test.

### Statistical table of the relative expression of IL-1β, IL-6 and TNF-α in the fracture end hematoma and serum samples under different bone mineral densities after fracture

The statistical results are as follows (Tables 4 and 5):

**Table 4:** Statistical table of serum immune factor levels in the osteoporotic and non-osteoporotic groups

Groups	n	Blood serum IL-1β	Blood serum IL-6	Blood serum TNF-α
		(ng/ml)	(μg/ml)	(ng/ml)
Osteoporosis group	29	16.74 ± 3.19	70.26 ± 6.27	804.31 ± 82.68
Non-osteoporosis group	19	11.53 ± 1.66	54.85 ± 7.41	569.54 ± 73.11
t		- 6.54	- 7.74	- 10.33
P		< 0.05	< 0.05	< 0.05

**Table 5:** The relative expression of immune factors in the fracture end hematoma samples in the two groups

Groups	n	Hematoma IL-1β	Hematoma IL-6	Hematoma TNF-α
Osteoporosis group	29	1.0366 ± 0.0294	1.0452 ± 0.0347	1.0336 ± 0.0371
Non-osteoporosis group	19	0.9577 ± 0.0267	0.9424 ± 0.0498	0.9470 ± 0.0252
t		- 9.61	- 7.84	- 9.63
P		< 0.05	< 0.05	< 0.05

According to the above data, the average levels of serum IL-1β, IL-6, and TNF-α and the average relative expression levels of IL-1β, IL-6, and TNF-α in the fracture end hematoma samples of the patients with osteoporotic fracture were higher than those in the non-osteoporotic group, and the differences were statistically significant (*P* < 0.05).

### Histograms of the relative expression of IL-1β, IL-6, and TNF-α in the serum and fracture end hematoma samples under different BMD conditions

**Fig. 1:**
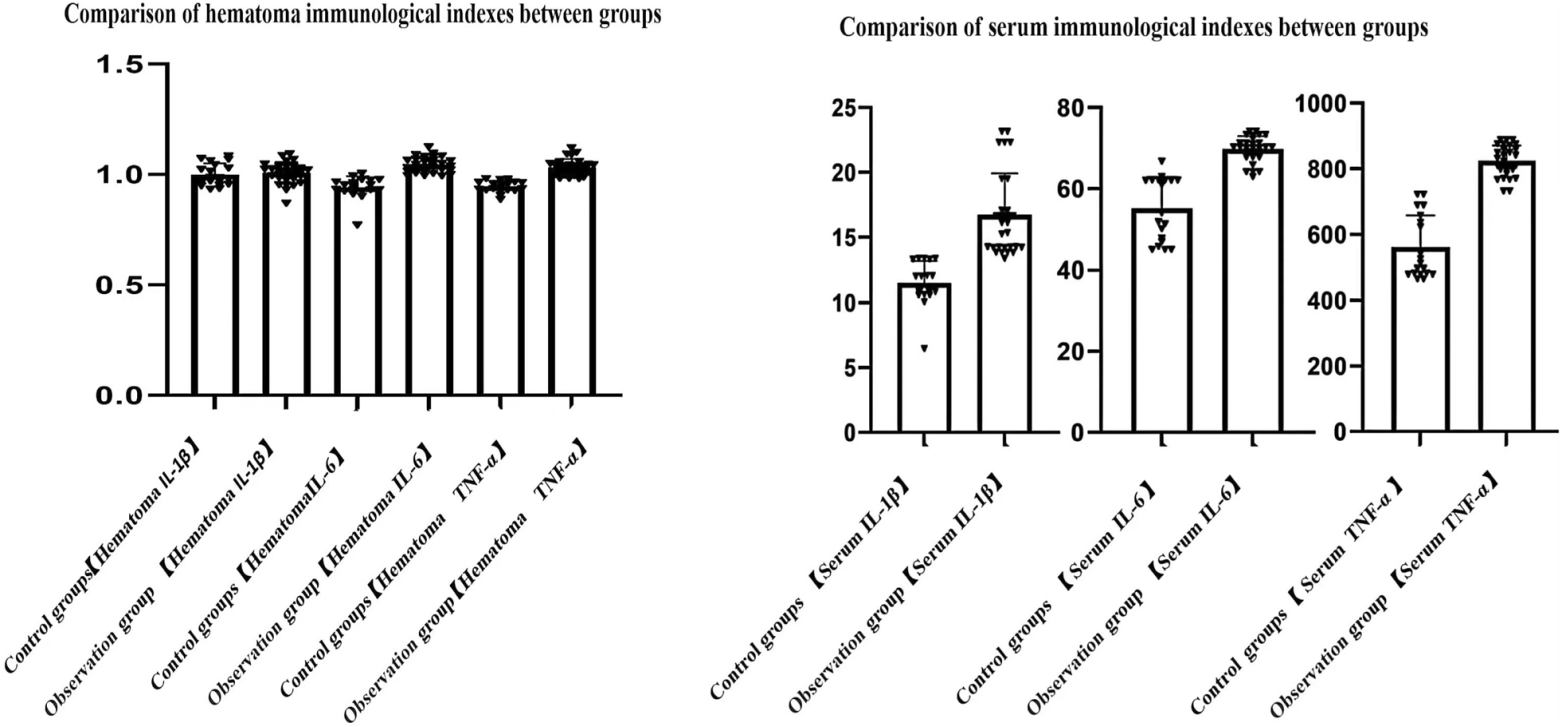
Immune factor levels in the fracture end hematoma samples of the osteoporosis and non-osteoporosis groups

The osteoporosis group was taken as the observation group, and the non-osteoporosis group was taken as the control group. The above data were grouped into histograms, as shown below (Fig. 1):

### Effect of active vitamin D3 on postoperative serum IL-1β, IL-6 and TNF-α levels in patients with osteoporotic distal radius fractures

Patients in the osteoporosis group were randomly divided into the treatment group and the control group. Both groups were given conventional fracture treatment and calcium carbonate D3 tablets supplementation, and the treatment group was additionally given oral active vitamin D3 treatment (Rogaecan, Roche, Switzerland; 0.25 μg/time, two times a day). Serum samples were collected on the seventh day after the operation to detect the levels of IL-1β, IL-6 and TNF-α.

### Statistical table of serum levels of IL-1β, IL-6, and TNF-α in patients with osteoporotic distal radius fractures after treatment with active vitamin D3

The serum immune factor levels of the treatment group and the control group were determined, and the independent sample t test method was used for statistical analysis. The results were as follows (Table 6):

**Table 6:** Serum immune factor levels in patients with osteoporotic fractures treated with active vitamin D3

Groups	n	After operation 7d	After operation 7d	After operation 7d
		Blood serum IL-1β	Blood serum IL-6	Blood serumTNF-α
		(ng/ml)	(µg/ml)	(ng/ml)
Treatment group	15	14.85 ± 1.82	53.72 ± 5.75	641.52 ± 101.51
Control group	14	16.62 ± 2.32	59.30 ± 6.54	721.85 ± 92.38
t		- 2.28	- 2.93	- 2.02
P		< 0.05	< 0.05	< 0.05

According to the above data, active vitamin D3 treatment affected the serum levels of IL-1β, IL-6 and TNF-α; specifically, the serum levels of the three immune factors in the treatment group were significantly lower than those in the control group on the seventh day after the operation (*P* < 0.05).

### Histograms of serum IL-1β, IL-6, and TNF-α levels in patients with osteoporotic distal radius fractures after active vitamin D3 treatment

**Fig. 2:**
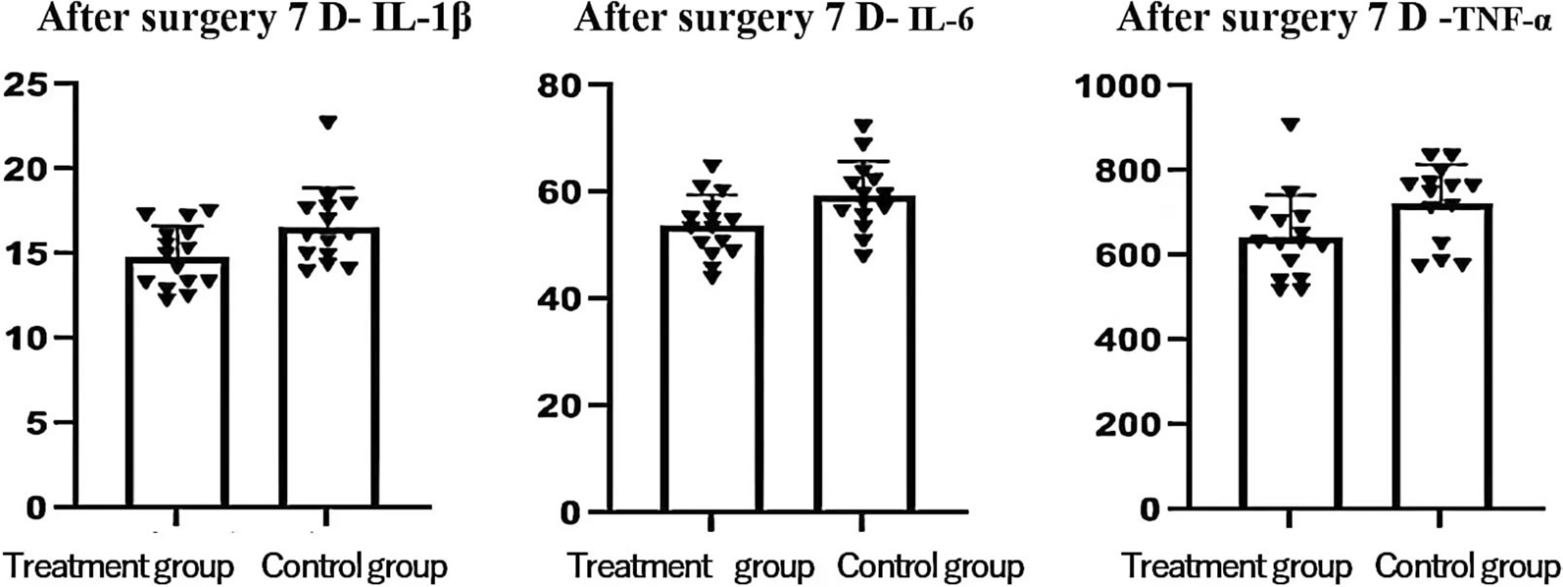
Histograms of serum immune factor levels after treatment with active vitamin D3

Based on the statistical results of the above data, histograms were generated to represent the serum levels of IL-1β, IL-6, and TNF-α after treatment of osteoporotic fractures with active vitamin D3, as shown below (Fig. 2).

### Effect of active vitamin D3 on the prognosis of patients with osteoporotic distal radius fractures

All 48 patients were followed up for 6 months. According to the GW wrist function score at 6 months after the operation, the wrist function of the patients was evaluated, and 19 cases were excellent, 28 cases were good, and 1 case was fair, with an excellent and good ratio of 98.1%. The average GW score was 3.15 ± 2.10, and the average healing time was 9.7 ± 1.3 weeks. The mean GW scores of the patients in the active vitamin D3 treatment group and the control group were statistically analyzed as follows (Table 7):

**Table 7:** Fracture prognosis between the active vitamin D3 treatment group and the control group

Groups	n	GW Score	Fracture healing time (weeks)
Treatment group	15	3.20 ± 1.47	9.7 ± 0.9
Control group	14	5.14 ± 1.56	10.6 ± 0.8
t		- 3.44	- 2.72
P		< 0.05	< 0.05

According to the above data, active vitamin D3 treatment significantly improved the wrist function of patients with osteoporotic distal radius fractures (*P* < 0.05). In addition, the fracture healing time of the active vitamin D3 treatment group was significantly shorter than that of the control group (*P* < 0.05).

### Typical cases

**Fig. 3:**
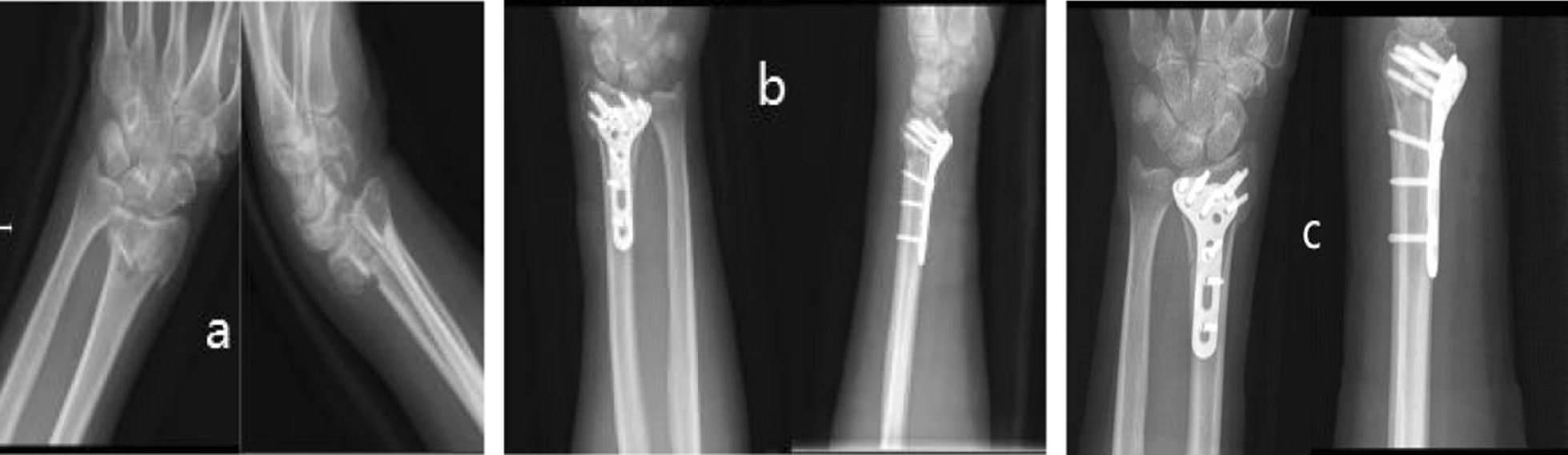
X-ray observations of Case 1. **a** Shows a preoperative X-ray showing a fracture of the right distal radius; **b** Shows the X-ray on the next day of surgery, showing the exact reduction and fixation of the fracture; **c** Shows the X-ray at 6 weeks after surgery with blurred fracture lines

**Case 1**: A 56-year-old female patient was diagnosed with left distal radius fracture. The preoperative BMD was T = -3.09. She was included in the osteoporosis group and the active vitamin D3 treatment group. The serum levels of IL-1β, IL-6 and TNF-α in this patient were 14.31 ng/ml, 67.00 μg/ml and 779.91 ng/ml, respectively. Open reduction and internal fixation of the left distal radius fracture were performed, and the hematoma at the fracture end was collected during the operation. Laboratory analysis showed that the relative expression levels of IL-1β, IL-6, and TNF-α in the hematoma at the fracture end were 1.0177, 1.0227, and 1.0095, respectively. Vitamin D3 was administered orally on the second day after surgery. The serum levels of IL-1β, IL-6 and TNF-α were 14.20 ng/ml, 57.20 μg/ml and 714.71 ng/ml on the seventh day after the operation. X-ray at 7 weeks after the operation showed a blurred fracture line. After 6 months of follow-up, the GW score of the wrist joint was 2, and the result was excellent (Fig. 3).

**Fig. 4:**
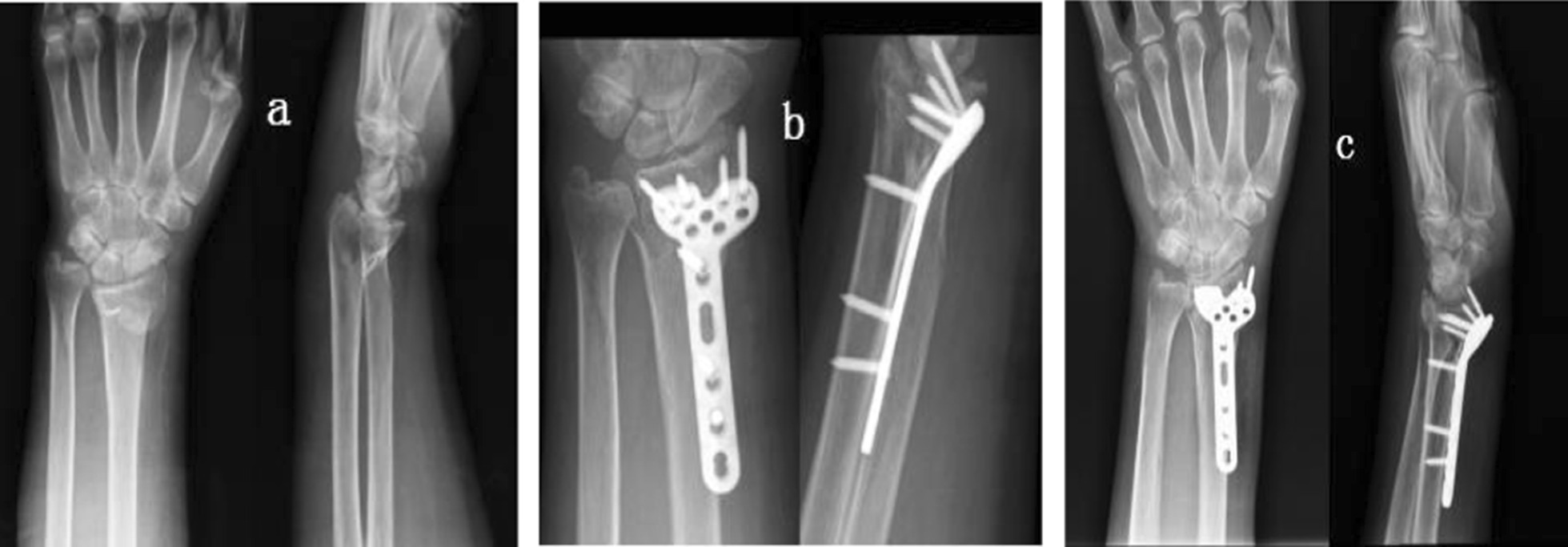
X-ray observations of Case 2. **a** Shows a preoperative X-ray showing a fracture of the right distal radius; **b** Shows the X-ray on the next day of surgery, showing the exact reduction and fixation of the fracture; **c** Shows the X-ray at 6 weeks after surgery, where the fracture line is still visible

**Case 2**: A 64-year-old female patient was diagnosed with a right distal radius fracture. The preoperative BMD test was T = -3.14, and she was included in the osteoporosis group and the control treatment group. The serum levels of IL-1β, IL-6 and TNF-α were 14.28 ng/ml, 66.11 µg/ml and 779.84 ng/ml, respectively. The right distal radius fracture was treated by open reduction and internal fixation, and the hematoma was collected during the operation. Laboratory analysis showed that the relative expression levels of IL-1β, IL-6, and TNF-α were 1.0177, 1.0227, and 1.0095, respectively. The serum levels of IL-1β, IL-6 and TNF-α were 13.97 ng/ml, 58.22 μg/ml and 718.47 ng/ml on the seventh day after the operation. Satisfactory fracture healing was achieved 11 weeks after the operation. After 6 months of follow-up, the GW score of the wrist joint was 5 points, which was good (Fig. 4).

## Discussion

At present, the academic community has reached a consensus that the content of immune factors has objective effects on osteoblasts (OB) and osteoclasts (OC) [10], but the specific effects of different inflammatory mediators on the activity of OBs and OCs are still being explored. IL-1β, IL-6, and TNF-α are common proinflammatory factors and are the most influential factors in the development of posttraumatic systemic inflammatory response syndrome (SIRS). In recent years, an increasing number of studies have explored the effect of the expression of these proinflammatory factors on the prognosis of fracture. Excessive expression of ILs often leads to difficulty in fracture healing. IL-1 is one of the most active inflammatory mediators in the body, and its main secreted form, IL-1β, is produced by immune cells such as macrophages. Il-1β is a major inflammatory factor in the IL-1 family that exercises biological functions [11]. Other studies have shown that the levels of IL-6 and IL-8 in patients with fracture nonunion are higher than those in patients with good fracture healing, and IL-8 and IL-6 are independent risk factors for postoperative fracture nonunion [12]. Some studies have shown that the prognosis of patients with traumatic fracture is closely related to the level of IL-6 [13]. The higher the serum level of IL-6 is, the worse the fracture prognosis and the higher the British Trauma Early Warning score [14]. In this study, the IL-1β and IL-6 levels in non-osteoporotic fracture patients were lower than those in osteoporotic fracture patients, and the prognosis was better. A consistent conclusion from the other direction, showing the possibility of proinflammatory factors for the clinical prediction of fracture prognosis. On the other hand, some scholars believe that the decline in bone in elderly individuals is related to the decline in immune factors with age. For example, an important role of IL-6 is to promote OC maturation while increasing RANKL expression [15]. In vitro experiments have also confirmed that the expression of OCs is increased by IL-6 stimulation, which affects bone metabolism [16].

TNF-α is a core inflammatory mediator that is usually produced after the body receives stimuli. It can directly or indirectly enhance the metabolic ability of the whole body, change hemodynamics [17], promote the anabolism of some other inflammatory mediators, and have different effects on fracture healing [18]. Some studies have found through animal experiments that the expression of IL-1β protein in the joint cavity of mice is significantly reduced after blocking TNF-α, suggesting that TNF-α is related to the production of IL-1β protein [19]. Similar conclusions have been reached in cell experiments, where MSU crystals can more effectively promote the synthesis of IL-1β precursor and IL-18 in medium supplemented with TNF-α [20]. TNF-α also has dual effects on fracture: on the one hand, it reduces new bone formation, and on the other hand, it enhances bone resorption, improves OC activity, and reduces cortical bone quality. Low concentrations of TNF-α combined with ILs can increase the migration of myogenic stem cells to the fracture end, while high concentrations of TNF-α have the effect of reducing bone formation [21]. In addition, TNF-α can enhance IL-17 and other immune factors, thereby promoting OC formation and bone resorption [22]. This study found that osteoporotic fracture patients had higher TNF-α levels and worse prognoses than non-osteoporotic fracture patients. Moreover, active vitamin D3 treatment significantly reduced serum TNF-α levels in patients with osteoporotic fractures. If we can further understand the influence of various immune factors on local bone metabolism, we can accurately predict the risk of fracture through the expression of immune factors and even improve the bone quality of patients with osteoporosis and the prognosis of patients with fracture by intervening in the immune status.

Vitamin D receptor can be expressed on T cells, B cells, macrophages and other immune cells and participate in the immune response. Studies have reported that active vitamin D3 can play a role in reducing the destruction of islet cells by autoinflammatory reactions, thereby maintaining the function of islet B cells [23]. The role of vitamin D in regulating calcium and phosphorus metabolism has been widely recognized by the medical community. In recent years, some scholars have reported a role of vitamin D in regulating immune factors and inhibiting the immune response. For example, active vitamin D can inhibit the activity of NF-kB in monocytes, thereby inhibiting the synthesis of IL-6 and TNF-α and improving the immune status [24]. The results of this study also showed that the levels of IL-1β, IL-6, and TNF-α in patients with osteoporotic fractures treated with calcium carbonate D3 tablets were lower than those in the control group, indicating that inflammation can be effectively controlled in patients with osteoporotic fractures treated with active vitamin D3. In addition, the mean healing time and the mean GW functional score at 6 months after surgery were better in the active vitamin D3 group than in the control group. Combined with the effect of vitamin D3 in promoting calcium absorption, it is speculated that active vitamin D3 treatment may further promote fracture healing through immune regulation. As a common vitamin D supplement, the effect of active vitamin D3 on osteoporosis has been widely recognized in clinical applications. These studies also indicated that active vitamin D3 could be used to effectively control the inflammatory response by regulating the expression of immune factors. After the occurrence of osteoporotic fracture, a patient's serum immunological indicators change, and the fracture end also has a strong immune response [25]. When exploring active vitamin D in the treatment of osteoporotic fractures, many previous studies have focused on the effect of vitamin D in promoting the absorption of calcium and phosphorus [26], thereby improving BMD and enhancing bone quality. Many positive conclusions have been obtained. The effect of active vitamin D3 treatment on improving BMD in patients with osteoporosis is now widely recognized in clinical practice. However, there are few studies on the effect of vitamin D on the prognosis of patients with osteoporotic fractures by regulating immune function. From the perspective of the effect of active vitamin D3 on the immune system and the prognosis of fractures, active vitamin D3 can reduce the levels of serum IL-1β, IL-6 and TNF-α and improve the prognosis of patients with osteoporotic fractures. Whether the ability of active vitamin D3 to promote calcium and phosphorus absorption and improve BMD is related to the synthesis and release of these immune factors needs to be further studied.

## Conclusion

After comparative analysis of serum immune factor levels and relative expression of immune factor in distal radius fracture end hematoma, the results suggested that the relative expressions of IL-1β, IL-6 and TNF-α in the hematoma of fracture end were positively correlated with the levels of corresponding immune factors in serum. Serum immunological indicators can be used to evaluate the immune status of the fracture end microenvironment.

The average levels of IL-1β, IL-6 and TNF-α in serum and fracture end hematoma samples of patients with osteoporotic fracture were higher than those in patients with non-osteoporotic fracture, suggesting that the systemic immune status and the immune status of the local microenvironment of the fracture site are changed when osteoporotic fracture occurs. The levels of IL-1β, IL-6 and TNF-α in patients with osteoporotic fractures were higher than those in patients with nonosteoporotic fractures.

Postoperative application of active vitamin D3 can promote a decrease in serum IL-1β, IL-6 and TNF-α levels in patients with osteoporotic fractures and improve the systemic immune status. Additionally, postoperative application of active vitamin D3 can improve wrist function and shorten fracture healing time in patients with osteoporotic distal radius fractures at 6 months after the operation. The combination of these two factors suggests that active vitamin D3 treatment can promote fracture healing and improve the symptoms of osteoporosis by affecting the levels of immune factors. Nevertheless, the mechanism needs further study.

## Data Availability

The datasets generated and/or analyzed during the current study are not publicly available due [REASON WHY DATA ARE NOT PUBLIC] but are available from the corresponding author on reasonable request.

## Abbreviations

OC: Osteoclast
OB: Osteoblast
Qpcr: Real-time quantitative PCR
IL: Interleukin
TNF: Tumor necrosis factor
SIRS: Systemic inflammatory response syndrome
